# Immediate postoperative of bariatric surgery in the intensive care unit *versus* an inpatient unit. A retrospective study with 828 patients

**DOI:** 10.5935/0103-507X.20170050

**Published:** 2017

**Authors:** Guilherme Loures de Araújo Penna, Igor Pedreira Vaz, Eduardo Côrtes Fonseca, Marcelo Kalichsztein, Gustavo Freitas Nobre

**Affiliations:** 1 Intensive Care Unit, Casa de Saúde São José - Rio de Janeiro (RJ), Brazil.; 2 Universidade Estácio de Sá - Rio de Janeiro (RJ), Brazil.

**Keywords:** Perioperative care, Quality of health care, Bariatric surgery/complications, Obesity/surgery, Intensive care units, Inpatient care units

## Abstract

**Objective:**

To compare the incidence of complications and the duration of hospitalization of patients undergoing bariatric surgery admitted to the intensive care unit or a post-surgical hospitalization unit.

**Methods:**

This retrospective observational study included 828 patients admitted between January 2010 and February 2015 during the immediate postoperative period of bariatric surgery in a hospital. Data were collected via electronic medical records. The Mann-Whitney test was used to compare continuous variables, and the chi-square was used to compare categorical variables.

**Results:**

Patients in both groups had similar demographic characteristics, with no significant differences in anthropometric data and comorbidities. There was no significant difference in the comparison of complications between the two groups. However, the group admitted to the intensive care unit had longer hospitalization times (median of 3 days *versus* 2 days, p < 0.05), and hospital costs were 8% higher.

**Conclusion:**

The present study found no benefit in the routine admittance of patients to the intensive care unit after undergoing bariatric surgery. This practice increased hospitalization time and hospital costs, which wasted resources. It is necessary to create objective criteria to identify patients requiring intensive care unit admission after bariatric surgery.

## Abstract (pt)

**Objetivo:**

Comparar a incidência de complicações e a duração da hospitalização de pacientes submetidos à cirurgia bariátrica internados na unidade de terapia intensiva ou de internação pós-cirúrgica.

**Métodos:**

Estudo observacional, retrospectivo, que incluiu 828 pacientes admitidos entre janeiro de 2010 e fevereiro de 2015 em pós-operatório imediato de cirurgia bariátrica em um hospital. Os dados foram coletados em prontuários eletrônicos. As variáveis contínuas foram comparadas utilizando-se o teste de Mann-Whitney e as categóricas, o qui quadrado.

**Resultados:**

Os pacientes dos dois grupos possuíam características demográficas semelhantes, sem diferença significativa dos dados antropométricos e comorbidades. Comparando-se as complicações entre os dois grupos, não houve diferença significativa. No entanto, o grupo admitido na unidade de terapia intensiva teve maior tempo de internação (mediana de 3 dias *versus* 2 dias; p < 0,05) e custo hospitalar 8% maior.

**Conclusão:**

O presente estudo não encontrou nenhum benefício na internação rotineira de pacientes submetidos à cirurgia bariátrica em unidade de terapia intensiva. Esta prática aumentou o tempo de internação e o custo hospitalar, desperdiçando recursos. É necessária a criação de critérios objetivos para identificar pacientes que necessitem de internação em unidade de terapia intensiva após cirurgia bariátrica.

## INTRODUCTION

Obesity is currently one of the major public health problems in Brazil and in the world and is an important risk factor for diseases with high prevalence and morbidity and mortality, such as systemic arterial hypertension, type 2 *diabetes mellitus*, dyslipidemia and atherosclerotic vascular disease, along with several types of malignant neoplasms, such as those of the breast, colon, prostate and liver.^(1-3)^

Bariatric surgery is the most recent and the most effective form of treatment for obesity.^(4-6)^ Currently, four different bariatric surgery techniques are approved in Brazil and are classified as restrictive, malabsorptive or mixed. For many years, the gastric *bypass* with Roux-en-Y reconstruction was considered the gold standard of bariatric techniques and the most accomplished procedure. However, in the past decade, vertical gastrectomy has proven to be an effective and simpler procedure to perform, which has led to an increase in its popularity. It is currently the most used surgical technique in the United States, performed in more than 42% of surgeries.^(7)^ The other modalities are vertical gastrectomy, adjustable gastric band and biliopancreatic diversion with duodenal switch. All of these procedures can be performed via videolaparoscopy or open surgery.

The enormous demand for efficient and long-lasting weight reduction therapies has generated great enthusiasm in relation to surgeries, with an impressive number of them performed each year. By 2013, more than 460,000 bariatric surgeries were performed worldwide, with more than 150,000 surgeries performed in the United States and Canada alone. In the same period, more than 86,000 procedures were performed in Brazil.^(8)^

Despite being an efficient approach in the treatment of obesity, bariatric surgeries have several possible postoperative complications, which can be divided into early and late complications. Early complications generate significant morbidity and elevations in hospitalization costs. Mortality related to bariatric surgeries remains below 1% and is approximately 0.3% in international studies.^(9-11)^ Among early complications, the most important include surgical wound infections, anastomosis dehiscence, fistulas, bleeding, venous thromboembolism and incisional hernias. The prevalence of postoperative complications is low, including bleeding in 0.5%, thromboembolism in 0.8% and operative wound complications in 1.8% of cases.^(9-11)^

Despite the increasing advances in the area, the decision to routinely admit patients who have undergone bariatric surgeries in an intensive care unit (ICU) is still controversial and is often performed based on subjective parameters or on the surgeon's experience.^(12-15)^ However, international studies show the current preference for avoiding routine ICU hospitalization, which reduces the mean hospital stay, reduces costs and releases beds, which are increasingly scarce, for other patients.^(8,16,17)^

The present study aimed to compare the incidence of complications and the duration of hospitalization of patients undergoing bariatric surgery admitted to the ICU or a post-surgical hospitalization unit. In addition, a comparative cost analysis of both units was performed.

## METHODS

This retrospective observational study included 828 patients admitted in a private hospital located in the city of Rio de Janeiro (RJ) between January 2010 and February 2015 in the immediate postoperative period of bariatric surgery. The study was approved by the local Ethics Committee under opinion 1.137.833 and consent term was waived.

Clinical and demographic data were collected from each patient's electronic medical records. The decision on the place of hospitalization of the patients included in the study was exclusively the responsibility of the professionals involved in the surgical procedure. Statistical comparisons of anthropometric data, comorbidities, complications, length of hospitalization and hospital costs were performed between patients admitted to the ICU or not during the immediate postoperative period. The Kolmogorov-Smirnov test was used to verify whether the variables were normally distributed. Continuous and categorical variables that were not normally distributed were compared using the Mann-Whitney test and the chi-square test, respectively.

Cost values were calculated using the absorption costing methodology. The final cost of hospitalization for each patient corresponds to the sum of the direct product (material and medication), the cost of the procedure (surgery and examinations) and the cost of the daily rate (hospital structure, such as personnel, equipment and indirect costs). Surgeons' fees were not included in this calculation.

The anthropometric data and comorbidities studied were age, gender, body mass index (BMI), systemic arterial hypertension, *diabetes mellitus*, hypothyroidism, severe asthma and obstructive sleep apnea with continuous positive airways pressure (CPAP). In addition to hospitalization time and hospital costs, the complications evaluated were death, sepsis, acute renal failure requiring dialysis, cardiovascular complications, unplanned intubation, need for tracheostomy, abdominal reoperation, percutaneous drainage, bleeding with need for blood transfusion, pneumonia, venous thromboembolism, fistula and anastomosis dehiscence.

## RESULTS

The mean age of the analyzed sample was 38 years, ranging from 16 to 70 years; 587 of the participants (70.93%) were female. Although bariatric surgeries were performed by 38 different surgeons, five surgeons were responsible for 625 procedures (75.5%).

Among the 828 surgeries performed in the period, 582 (70%) were by video laparoscopic gastric bypass, while another 216 (26%) were by video laparoscopic gastric sleeve. There were also 20 procedures of gastric bypass (2.5%) and 4 of gastric sleeve performed by laparotomy (0.5%). There was one death during the surgical period - a patient who underwent the video laparoscopic *bypass* technique - due to massive pulmonary thromboembolism.

A total of 319 individuals (38.52%) were admitted to the ICU immediately after surgery, while 509 (61.48%) were referred to the postoperative unit. There were no statistically significant differences between the groups in either the anthropometric data or the comorbidities surveyed (Table 1). When evaluating the presence of multiple comorbidities, no statistically significant differences between the two groups were found (Table 2).

**Table 1:** Anthropometric and comorbidities data (N = 828)

Immediate postoperative in the ICU	Yes (N = 319)	No (N = 509)
Age	39.05 ± 10.50	38.38 ± 10.39
Gender (male)	93 (29.25)	148 (29.2)
BMI (kg/m^2^)	41.68 ± 5.01	41.93 ± 5.19
BMI > 50 kg/m^2^	23 (7.21)	37 (7.26)
Diabetes	65 (20.44)	110 (21.5)
Hypothyroidism	25 (7.86)	32 (6.27)
Hypertension	148 (46.54)	232 (45.49)
Severe asthma	4 (1.26)	13 (2.55)
Sleep apnea using CPAP	9 (2.83)	11 (2.16)
Length of hospital stay (days)*	3.00 [3.00 - 4.00]	2.00 [2.00 - 3.00]

ICU - intensive care unit; BMI - body mass index; CPAP - continuous positive airway pressure.

* p < 0.05. Results expressed as numbers (%), means ± standard deviations or medians [25 - 75^th^ percentiles].

**Table 2:** Number of comorbidities by immediate postoperative destination

Immediate postoperative in the ICU	Yes (N = 318)	No (N = 510)
No comorbidities	145 (45.60)	227 (44.51)
One comorbidity	104 (32.70)	180 (35.29)
Two or more comorbidities	69 (21.70)	103 (20.20)

ICU - intensive care unit.

* p < 0.05. The results are expressed as numbers (%).

The comparison of the complications between these two groups showed no significant differences in any of the parameters analyzed, including the total complications (Table 3). However, in the group sent to the ICU, a greater median number of days of hospitalization (3 [2.00 - 4.00] *versus* 2 [2.00 - 3.00]; p < 0.05) and an 8% higher hospital cost (R$ 25,063.57 [23,195.41 - 27,595.20] *versus* R$ 23,237.33 [21,493.98 - 25,796.81]; p < 0.05) were observed in relation to the other patients.

**Table 3:** Complications by immediate postoperative destination

Immediate postoperative in the ICU	Yes (N = 318)	No (N = 510)
Sepsis	1 (0.31)	4 (0.79)
Acute renal failure	0 (0)	3 (0.59)
Cardiovascular complications	0 (0)	0 (0)
Deaths	1 (0.31)	0 (0)
Reintubation	2 (0.63)	4 (0.79)
Tracheostomy	0 (0)	2 (0.39)
Percutaneous drainage	1 (0.31)	4 (0.79)
Reoperation	12 (3.76)	11 (2.16)
Bleeding	8 (2.51)	6 (1.18)
Pneumonia	0 (0)	0 (0)
Venous thromboembolism	1 (0.31)	2 (0.39)
Fistula	4 (1.25)	9 (1.77)
Suture dehiscence	6 (1.88)	8 (1.57)
Any complications	14 (4.40)	13 (2.54)

ICU - intensive care unit. The results are expressed as number (%).

* p < 0.05.

## DISCUSSION

The findings of the present study did not show a difference between patients admitted to ICU beds and those who were not admitted to the ICU with regard to anthropometric data, previous comorbidities and postoperative complications.

This study had death rates and complications close to those of most national and international reports; the only death occurred due to massive pulmonary embolism.^(6,8,18,19)^ Hutter et al. conducted a study using data from the American College of Surgeons involving 28,616 patients operated upon in 109 different hospitals, and the mortality rate was 0.12%, i.e., the same rate verified in the current study.^(10)^

**Figure 1:**
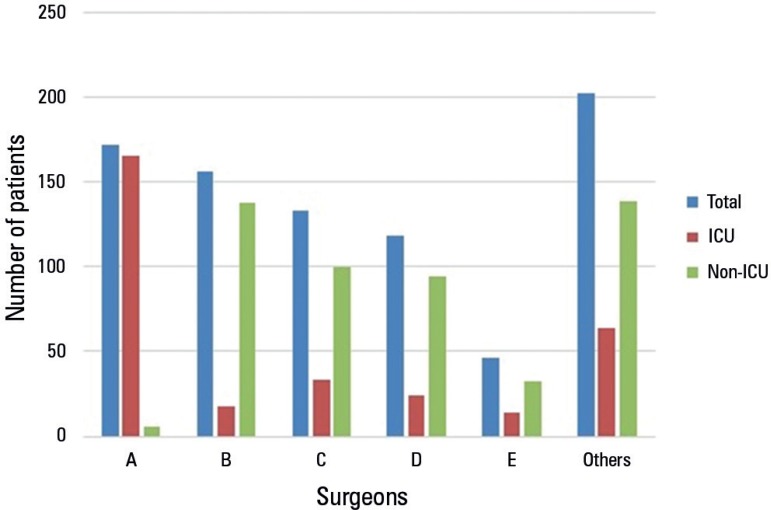
Number of surgeries per surgeon. ICU - intensive care unit.

In the present study, there were no significant differences in anthropometric data and comorbidities between the groups, indicating that these variables were not criteria used to support the decision of whether or not a patient should be admitted to the ICU. The analysis of in-hospital complications also showed no statistically relevant differences, which weakens the hypothesis that patients hospitalized in the ICU had significant intraoperative complications. Furthermore, we observed that the percentage of patients hospitalized in the immediate postoperative period in the ICU of each surgeon differed significantly (Figure 1) and that among the professionals with larger numbers of procedures, there were no statistically significant differences in the complication rates. Therefore, subjective parameters of each professional seem to have guided this important decision.

In international studies, the rates of immediate ICU admission are significantly lower than in the present study, not exceeding 8% and are justified mainly by risk factors related to old age, BMI greater than 50kg/m^2^ or intraoperative complications.^(12,13,16,17)^ Research involving 12,062 patients undergoing bariatric surgeries, of whom only 590 (4.90%) were admitted to the ICU postoperatively, showed that this group of patients had a significantly higher mean age and more frequently required an abdominal reoperation. Thus, contrary to what was verified in the present study, both groups had different characteristics, and ICU stay was better justified.^(16)^ A prospective Brazilian study with a sample of 120 patients submitted to laparoscopic bariatric surgery tested an index to evaluate the need for immediate hospitalization in intensive care, with no patients admitted to the ICU and no occurrence of postoperative complications.^(14)^

The incidence rates of bleeding with need for blood transfusion and abdominal reoperation in patients operated upon by one of the five professionals who performed the most bariatric surgeries at the São José Health House during the study period were significantly lower in relation to the rates of the other complications (Table 4). The greater experience of surgeons in this type of procedure seems to be an important predictor of a good prognosis. A Norwegian study showed no significant differences in the rates of complications among surgeons who performed at least 100 surgeries but also showed reductions in the duration of hospitalization and the procedure, indicating that the surgeon's learning curve may influence the postoperative results.^(20)^

**Table 4:** Complications by surgeon

	A (N = 172)	B (N = 156)	C (N = 133)	D (N = 118)	Others (N = 249)
Sepsis	1 (0.58)	0 (0)	0 (0)	0 (0)	4 (1.60)
Acute renal failure	1 (0.58)	0 (0)	0 (0)	0 (0)	2 (0.80)
Cardiovascular complication	0 (0)	0 (0)	0 (0)	0 (0)	0 (0)
Reintubation	1 0.58)	0 (0)	0 (0)	0 (0)	5 (2.00)
Tracheostomy	1 (0.58)	0 (0)	0 (0)	0 (0)	1 (0.40)
Percutaneous drainage	1 (0.58)	0 (0)	0 (0)	0 (0)	4 (1.60)
Reoperation	4 (2.32)	2 (1.28)	3 (2.25)	1 (0.85)	13 (5.22)*
Bleeding	3 (1.74)	1 (0.64)	0 (0)	0 (0)	10 (4.01)*
Pneumonia	0 (0)	0 (0)	0 (0)	0 (0)	0 (0)
Venous thromboembolism	1 (0.58)	1 (0.64)	0 (0)	0 (0)	1 (0.40)
Fistula	2 (1.16)	2 (1.28)	2 (1.50)	0 (0)	7 (2.81)
Suture dehiscence	3 (1.74)	2 (1.28)	2 (1.50)	0 (0)	7 (2.81)
Death	1 (0.58)	0 (0)	0 (0)	0 (0)	0 (0)

* p < 0.05. The results are expressed as numbers (%).

The present study has limitations, mainly related to its retrospective design, and the data analyzed were collected by several different professionals. This study was conducted in a single center, and there may be local selection bias, determined by the fact that most surgical procedures were performed by only a few professionals. In addition, the reduced incidence of complications inherent to bariatric surgeries generated a low total number of complications in the study, which may have influenced the achievement of statistically significant results. However, we are not aware of any other national study that has approached this subject with such a large number of patients. Finally, the study was limited to analyzing hospitalizations for bariatric surgery, excluding late complications and readmissions.

## CONCLUSION

There were no significant differences in the rates of postoperative complications between patients admitted to the intensive care unit or not, with increases only in hospitalization time and total cost in the group hospitalized for intensive care. The lack of evidence regarding the benefits of routine hospitalization in an intensive care unit immediately post-bariatric surgery shows the need to create objective criteria that are scientifically validated for the determination of this conduct. Unnecessary hospitalization in an intensive care unit increases the costs of hospitalization and postpones the patient's discharge and return to their daily activities, in addition to occupying a bed that could be used by another patient.
